# Fabrication of an Anatomically Realistic Intestinal Phantom with Villous Microstructure

**DOI:** 10.3390/bioengineering13080943

**Published:** 2026-08-21

**Authors:** Rohit Dey, Jiaming Du, Theodore Mah, Jack Shanks, James Hacunda, Savo Topic, Safak Yalcin, Cheng Yang, Yihao Zheng

**Affiliations:** 1 Department of Mechanical and Materials Engineering, Worcester Polytechnic Institute, 100 Institute Rd., Worcester, MA 01609, USAyzheng8@wpi.edu (Y.Z.); 2 Takeda Development Center Americas, Inc., 500 Kendall Street, Cambridge, MA 02142, USAcheng.yang@takeda.com (C.Y.)

**Keywords:** intestinal phantom, villous microstructure, video capsule endoscopy (VCE), celiac disease, PolyJet 3D printing, additive manufacturing

## Abstract

The accurate evaluation of gastrointestinal (GI) diseases such as celiac disease (CeD) relies on the assessment of villous architecture, yet progress in imaging-based diagnostics, particularly video capsule endoscopy (VCE), is constrained by the absence of anatomically realistic and reproducible physical models of the intestinal mucosa. Existing benchtop phantoms typically reproduce gross luminal curvature but fail to capture the sub-millimeter villous microstructure, the optical scattering behavior, and the luminal folding of native mucosa that together shape its endoscopic appearance. We developed a modular fabrication framework for an anatomically realistic small intestinal phantom with controlled villous microstructure. High-resolution drop-on-demand photopolymer material jetting was used to print discrete patches of villous-like micropillar arrays with tunable height, diameter, and spacing parameterized from histological data spanning Marsh 0 to 3c classifications. The printed patches were then dyed for mucosal-color realism, bonded onto a polyester–spandex substrate, rolled into a continuous tube, and shaped with adjustable retainer rings to introduce luminal folds. Optical microscopy confirmed dimensional fidelity within ±10% of design values with patch-to-patch variation below 7%, and VCE imaging of healthy and atrophic configurations achieved structural similarity (SSIM) values of 0.625 and 0.761 against clinical mucosal imagery. This reproducible platform supports VCE device validation, imaging dataset generation, and clinician training in gastrointestinal imaging.

## 1. Introduction

**Figure 1:**
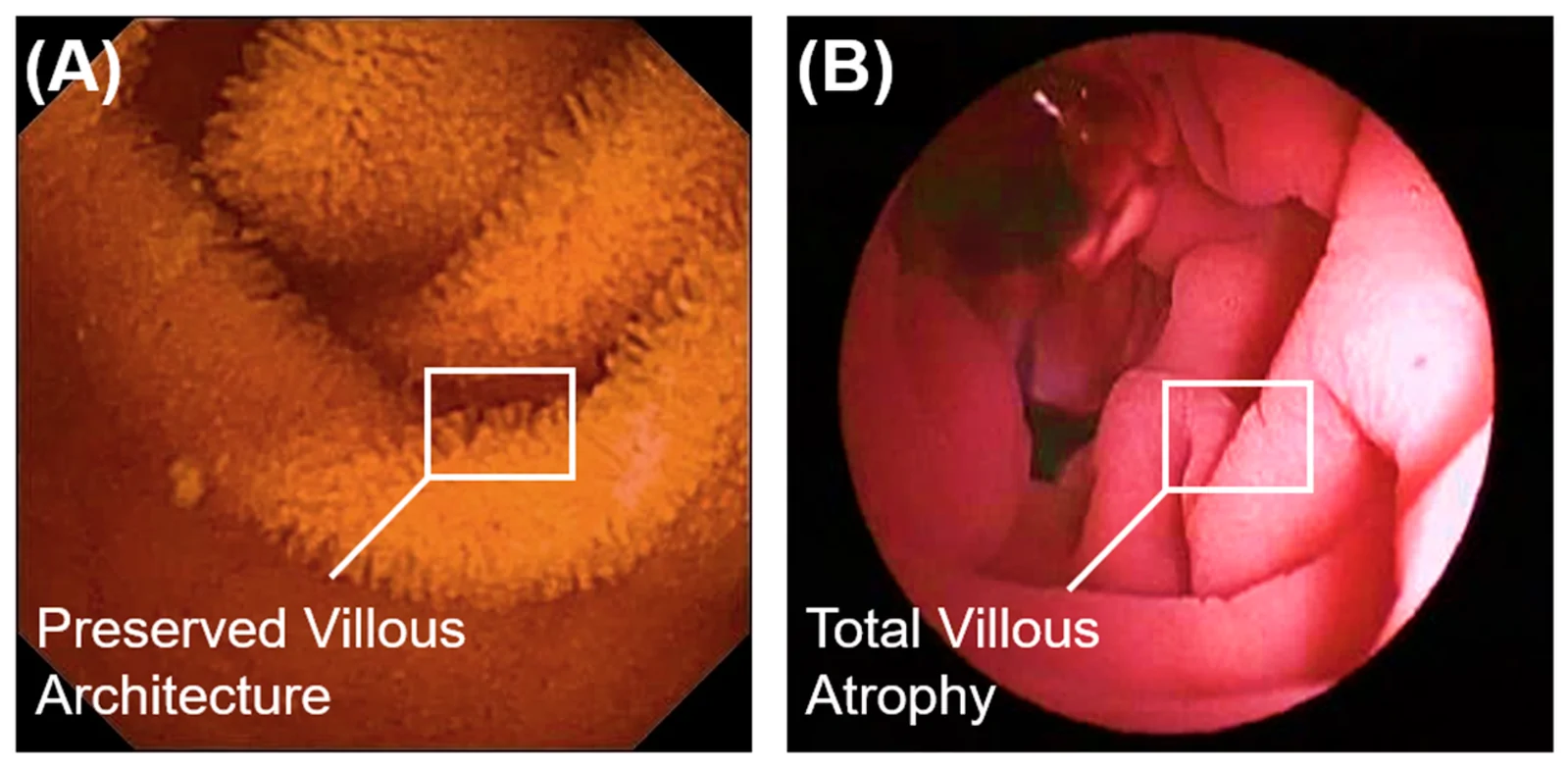
Endoscopic view of small intestinal mucosa. (**A**) Healthy mucosa with well-defined villous architecture and surface texture. (**B**) Flattened surface indicative of villous atrophy in celiac disease.

The accurate visualization of the small intestinal mucosa is vital for evaluation and characterization of a range of gastrointestinal (GI) disorders, including celiac disease (CeD), Crohn’s disease, and inflammatory enteropathies. These diseases are characterized by alterations in villous architecture, particularly villous atrophy, crypt hyperplasia, and mucosal flattening, which are linked to impaired nutrient absorption and compromised mucosal function [1]. Figure 1 illustrates representative endoscopic images demonstrating features of villous atrophy alongside normal mucosal architecture. The villous surface architecture of the small intestinal mucosa, composed of sub-millimeter ridges and folds, plays an important role in shaping tissue surface topology and governs optical scattering, mechanical compliance, and absorptive surface area. Alterations in villous architecture associated with tissue properties serve as structural tissue biomarkers that reflect mucosal injury during disease progression [2,3].

Video capsule endoscopy (VCE) provides minimally invasive visualization of the small intestine, which is difficult to access with conventional endoscopy due to its long length and complex looping. VCE has proven effective in detecting mucosal abnormalities, but quantitative analysis and algorithmic interpretation remain limited by the lack of anatomically realistic, reproducible testbeds that can replicate the complex micro-texture of real intestinal mucosa [4,5]. Most validation studies for VCE- and AI-based lesion detection rely on clinical datasets that exhibit variability in illumination, orientation, and tissue motion, making standardization and benchmarking difficult [6]. Deep-learning models have demonstrated strong performance for VCE-based celiac and lesion detection [7,8,9], yet their development and fair benchmarking remain constrained by the availability of standardized, well-annotated data.

**Figure 2:**
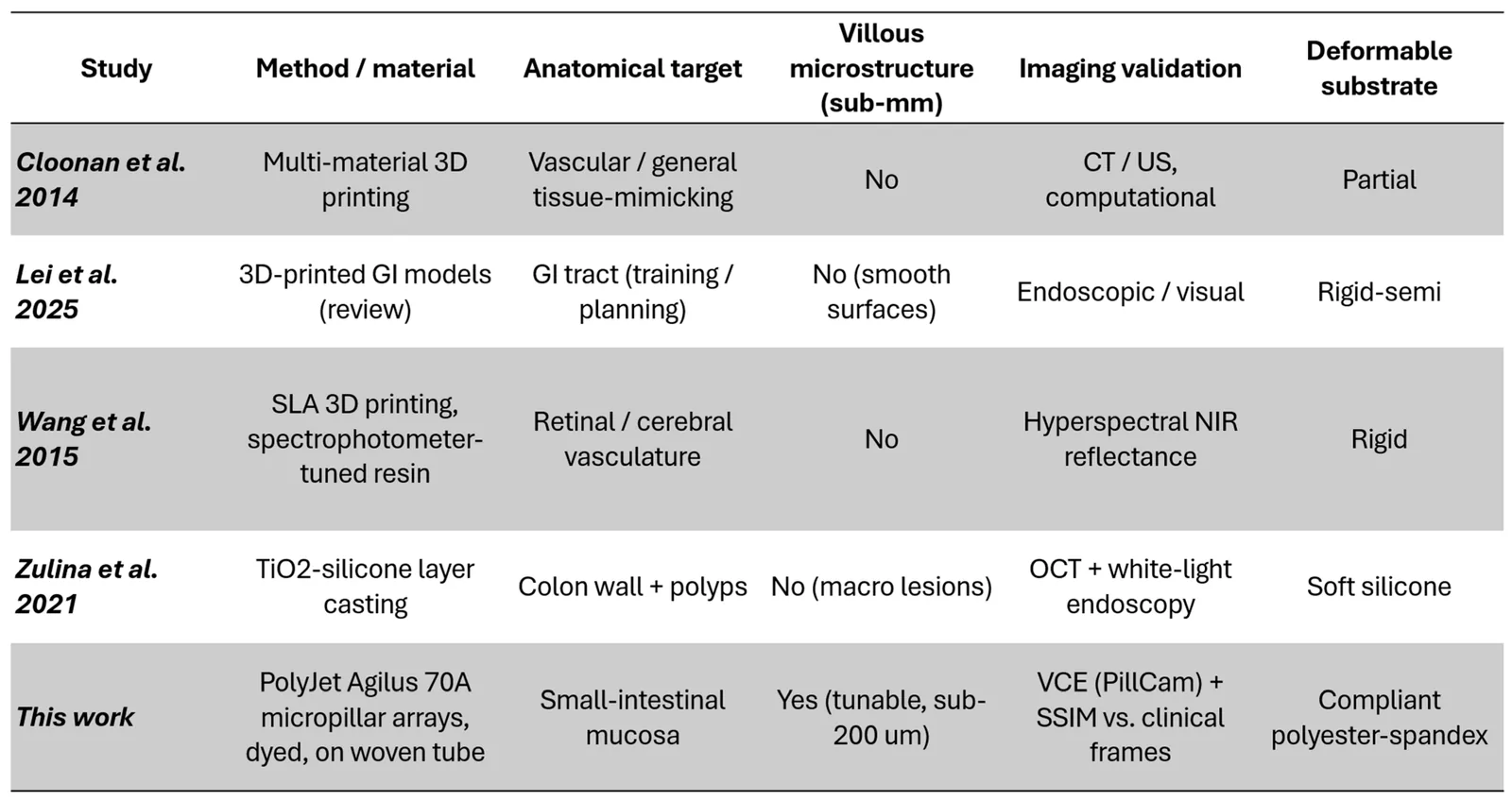
Comparison of representative intestinal and gastrointestinal phantoms against capabilities relevant to VCE realism [10,13,14,15].

Recent studies have explored the use of in vitro phantoms and computational reconstructions to replicate intestinal morphology [10,11,12]. However, most existing benchtop models—typically fabricated from silicone or hydrogel—capture only the gross curvature of the intestinal wall and fail to reproduce the fine villous microstructure that governs optical scattering and mechanical compliance. Even advanced training models exhibit smooth, featureless surfaces and lack the subsurface light diffusion and depth-dependent reflectivity characteristic of real mucosa [13,14,15]. Hydrogel-based tissue surrogates have achieved partial optical realism, but their microfeatures are poorly defined, and their mechanical properties do not match those of hydrated intestinal tissue. These limitations reduce their usefulness for video capsule endoscopy (VCE) validation, optical calibration, and algorithmic training, underscoring the need for a phantom that combines both microstructural fidelity and deformability. Figure 2 summarizes representative intestinal and gastrointestinal phantoms alongside the structural, optical, and validation capabilities most relevant to capsule-endoscopy realism, positioning the present work against the current state of the art.

**Figure 3:**
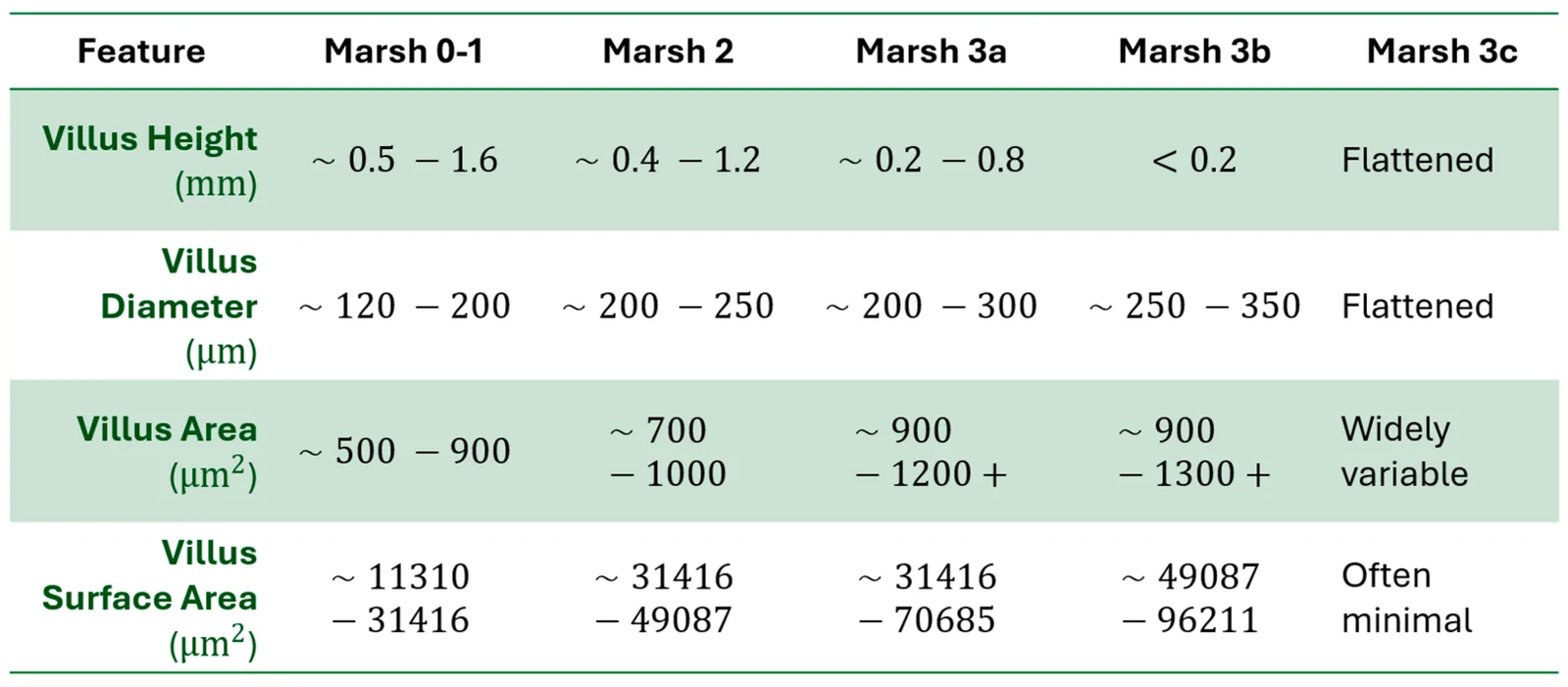
Morphometric parameters of villous morphology across Marsh classifications. Villus height, diameter, area, and surface area compiled from histological literature for Marsh 0–3c.

**Figure 4:**
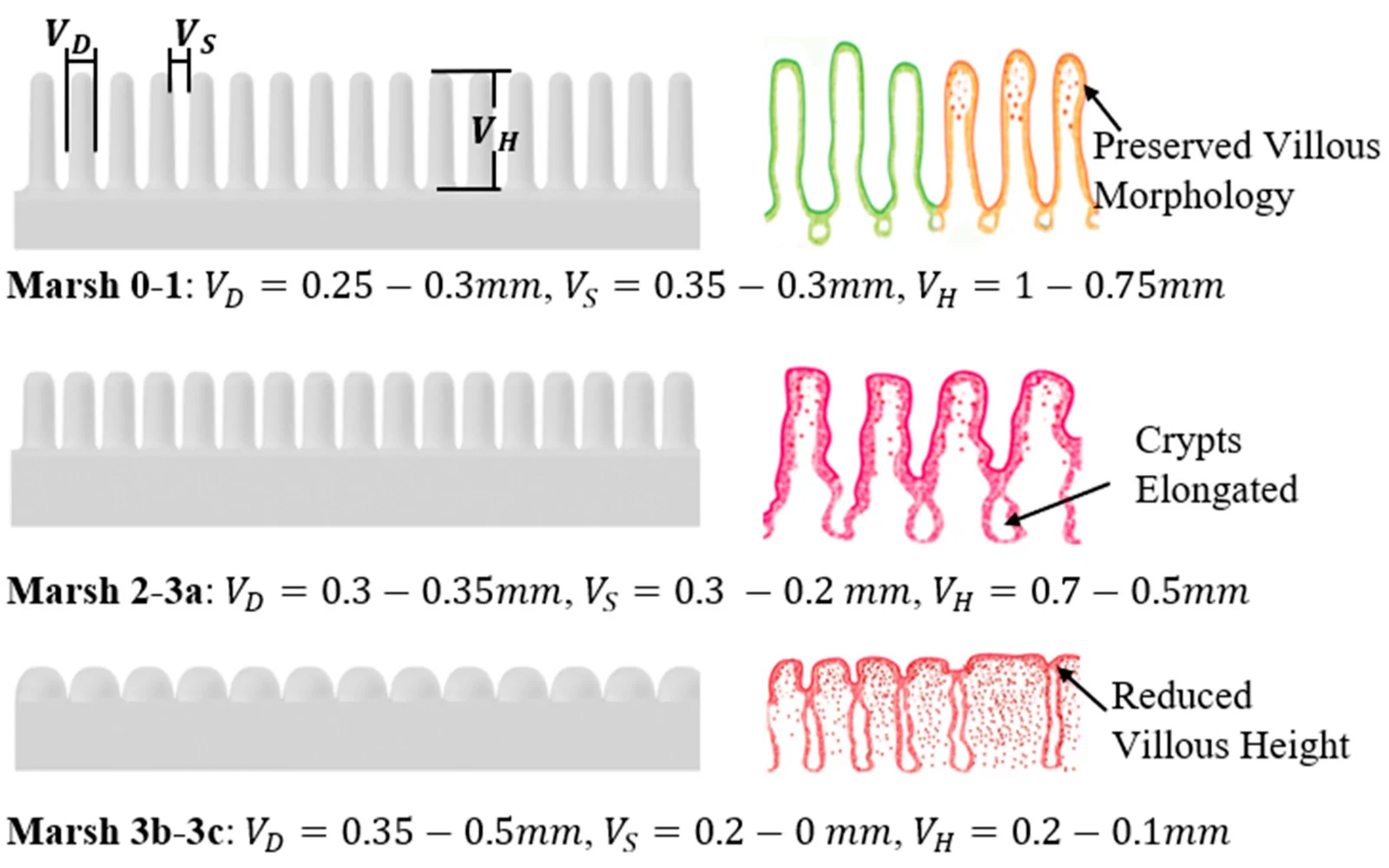
CAD models of villous microstructure across Marsh stages. Parametric designs show decreasing villus height ($V_{H}$) and spacing ($V_{S}$) with increasing diameter ($V_{D}$), from healthy (Marsh 0–1) to atrophic (Marsh 3b–3c).

To address these limitations, this study introduces a modular fabrication framework for creating anatomically realistic small intestinal phantoms that integrate microstructural fidelity with anatomically realistic luminal curvature and folding. The approach combines high-resolution photopolymer material-jetting with a compliant woven substrate to produce a soft, deformable construct that reproduces the villous microstructure, surface geometry, and endoscopic visual appearance of intestinal mucosa. Quantitative histologic measurements reported in the literature (Figure 3), including villus height, crypt depth, and established morphometric descriptors associated with Marsh classifications, informed the development of parametric CAD models (Figure 4), capturing the progression from healthy elongated villous architecture (Marsh 0–1) to flattened atrophic surfaces (Marsh 3b–3c) [10,16,17]. By integrating these data-driven design principles with an adaptable patch-based fabrication process, the resulting phantom reliably reproduces villous microstructure, luminal folds, and the mucosal appearance captured under endoscopic imaging. Discrete patches representing different disease stages can be independently fabricated and assembled in arbitrary spatial arrangements, allowing the framework to support user-defined disease distributions, scalable phantom lengths, and extension to other tubular mucosal tissues. Together, these capabilities establish a standardized, reproducible platform for optical benchmarking, VCE device validation, and the generation of high-quality imaging datasets to support clinician training and the AI-based evaluation and characterization of GI disorders.

## 2. Materials and Methods

**Figure 5:**
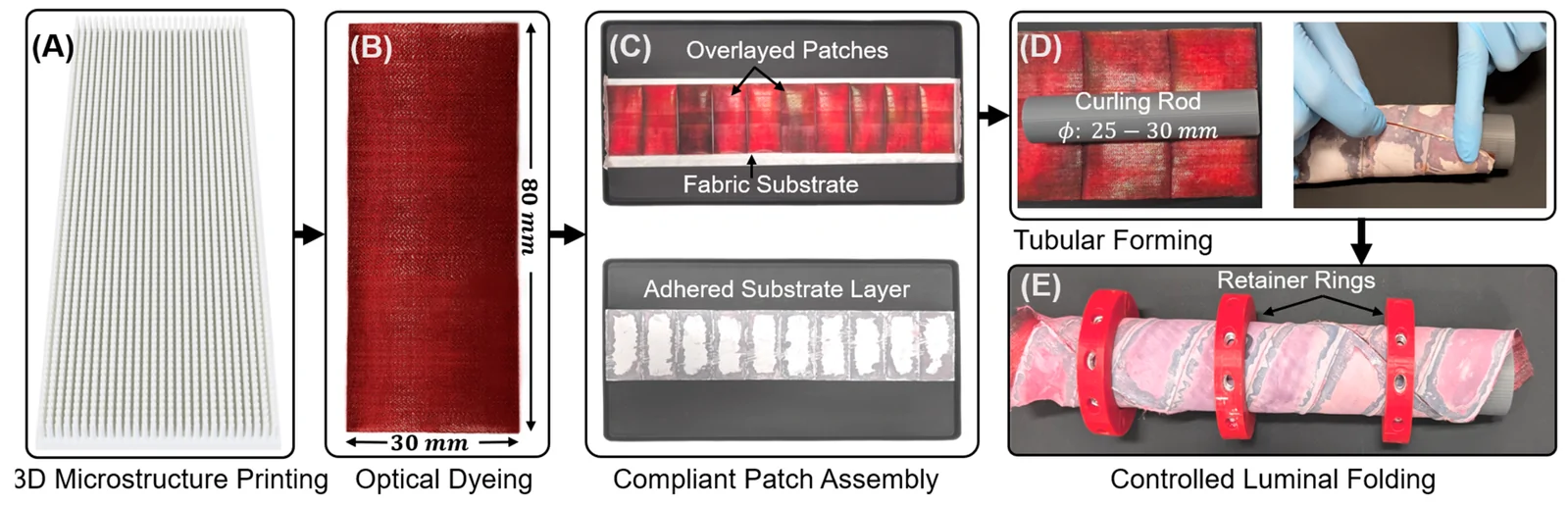
Fabrication workflow of the intestinal phantom. (**A**) Villous-array printing, (**B**) optical dyeing, (**C**) patch assembly on the fabric substrate, (**D**) tubular forming around a rod, and (**E**) luminal folding with adjustable retainer rings.

Development of the intestinal phantom followed a modular fabrication workflow combining quantitative histological modeling, high-resolution 3D printing, and compliant structural assembly. The overall process, summarized in Figure 5, comprised five sequential stages: 3D microstructure printing, optical dyeing, compliant patch assembly, tubular forming, and controlled luminal folding.

### 2.1. Data-Driven Design and Parametric CAD Modeling

Histological metrics (Figure 3) guided the design of villous microstructures across disease severities. The villous dimensions used to parameterize the CAD models were compiled from a cumulative review of published histological studies reporting the quantitative morphometry of small intestinal mucosa across Marsh stages [16,17,18,19], rather than from any single database. Staging follows the Marsh grading scheme [20] as modified by Oberhuber et al. to subdivide the Marsh 3 lesion into 3a–3c categories [21]. Villus height ($V_{H}$), base diameter ($V_{D}$), and spacing ($V_{S}$) were parameterized for five configurations ranging from healthy (Marsh 0–1; 1.0 mm height, 0.25 mm diameter, 0.35 mm spacing) to severely atrophic (Marsh 3c; 0.1 mm height, 0.5 mm diameter, 0 mm spacing). Figure 4 illustrates the CAD models representing these morphologies.

Villus arrays were modeled in SolidWorks 2025 as periodic lattices on 80 × 30 mm rectangular plates, each representing a discrete mucosal patch. Parametric modeling allowed systematic variation to simulate disease severity. Five primary configurations to represent the continuum from healthy to fully atrophic tissue are as follows:

- Healthy (Marsh 0): 1.0 mm height, 0.25 mm diameter, 0.35 mm spacing.
- Intermediate 1: 0.75 mm height, 0.30 mm diameter, 0.30 mm spacing.
- Intermediate 2 (Marsh 3a): 0.50 mm height, 0.30 mm diameter, 0.20 mm spacing.
- Atrophic (Marsh 3b): 0.20 mm height, 0.35 mm diameter, 0.20 mm spacing.
- Severe Atrophy (Marsh 3c): 0.10 mm height, 0.50 mm diameter, 0 mm spacing

### 2.2. Microstructure Printing and Conditioning

STL models were printed on a Stratasys J826 PolyJet (Stratasys Ltd., Minnetonka, MN, USA) system in a translucent flexible photopolymer produced by blending Agilus30 with Vero to a Shore A 60–70 durometer (14–16 µm layer thickness); this blended digital material is referred to hereafter as “Agilus 70A” for its ~70 Shore A hardness. The material was selected for reasons tied to the phantom’s imaging purpose: its translucency provides the subsurface light diffusion that underlies optical realism, and its low durometer keeps the printed patches flexible enough to roll into the tubular geometry and conform to the folds induced by the retainer rings, so that the assembled phantom presents realistic curvature and luminal folds under the capsule [22]. All patches were printed in a single, fixed orientation, with the 80 × 30 mm substrate plane laid parallel to the build tray (XY plane) and the villous micropillar axes aligned with the build direction (+Z), so that each pillar was formed by the successive deposition of layers along its long axis. This orientation was held constant across all Marsh-stage configurations to standardize feature fidelity and to avoid the anisotropy and staircase effects that an in-plane orientation would introduce, since build orientation is known to affect the dimensional accuracy of fine features in PolyJet material jetting [23].

The modular, patch-based build also defines the fabrication throughput. The ten 80 × 30 mm patches forming one representative phantom were printed together in a single build, requiring approximately 85–90 min on average. Because material jetting deposits each layer across the full tray simultaneously, total print time is governed primarily by build height and is largely independent of the number of patches per layer, so the ten-patch build corresponds to roughly 8.5–9 min per patch. The patch count per phantom is not fixed and can be increased to extend phantom length, with print time scaling by the number of build trays rather than linearly with patch count. These times exclude the post-processing steps described below.

After printing, patches were conditioned through a three-step sequence of support removal, dyeing, and drying. Support material was first removed, after which each patch was immersed in a warm scarlet-dye bath for 24 h to achieve a reddish, translucent tone visually similar to intestinal mucosa (Figure 5B). Patches were then air-dried gradually at room temperature before assembly, with drying controlled to prevent dimensional distortion of the fine villous features.

Because the resin’s inherent properties determine not only initial performance but also durability, its behavior over time warrants consideration. As with all photopolymers, material properties evolve with aging: continued crosslinking under ambient ultraviolet exposure, moisture uptake, and thermal cycling can alter mechanical response and dimensional stability, effects documented for the Agilus/Vero family and for PolyJet materials more broadly [24,25]. Long-term stability, and its mitigation through controlled storage and surface protection, is therefore identified as a translational consideration and a target for future characterization.

### 2.3. Compliant Patch Assembly and Tubular Forming

After dyeing, patches were adhered edge-to-edge to a polyester–spandex substrate, forming a flexible composite sheet (Figure 5C). The sheet was rolled around a 25–30 mm diameter rod and sealed longitudinally with silicone RTV adhesive to create a continuous tube (Figure 5D). This step established the primary intestinal geometry while maintaining elasticity and curvature. The polyester–spandex substrate was selected to provide the macro-scale elastic deformability required for tube forming, luminal curvature, and retainer-ring folding, decoupling this bulk flexibility from the microstructural fidelity of the printed villous layer. It was not intended to reproduce the stress–strain response of the intestinal wall; a quantitative mechanical comparison against native tissue is deferred to future work.

### 2.4. Topological Shaping and Retainer Rings

**Figure 6:**
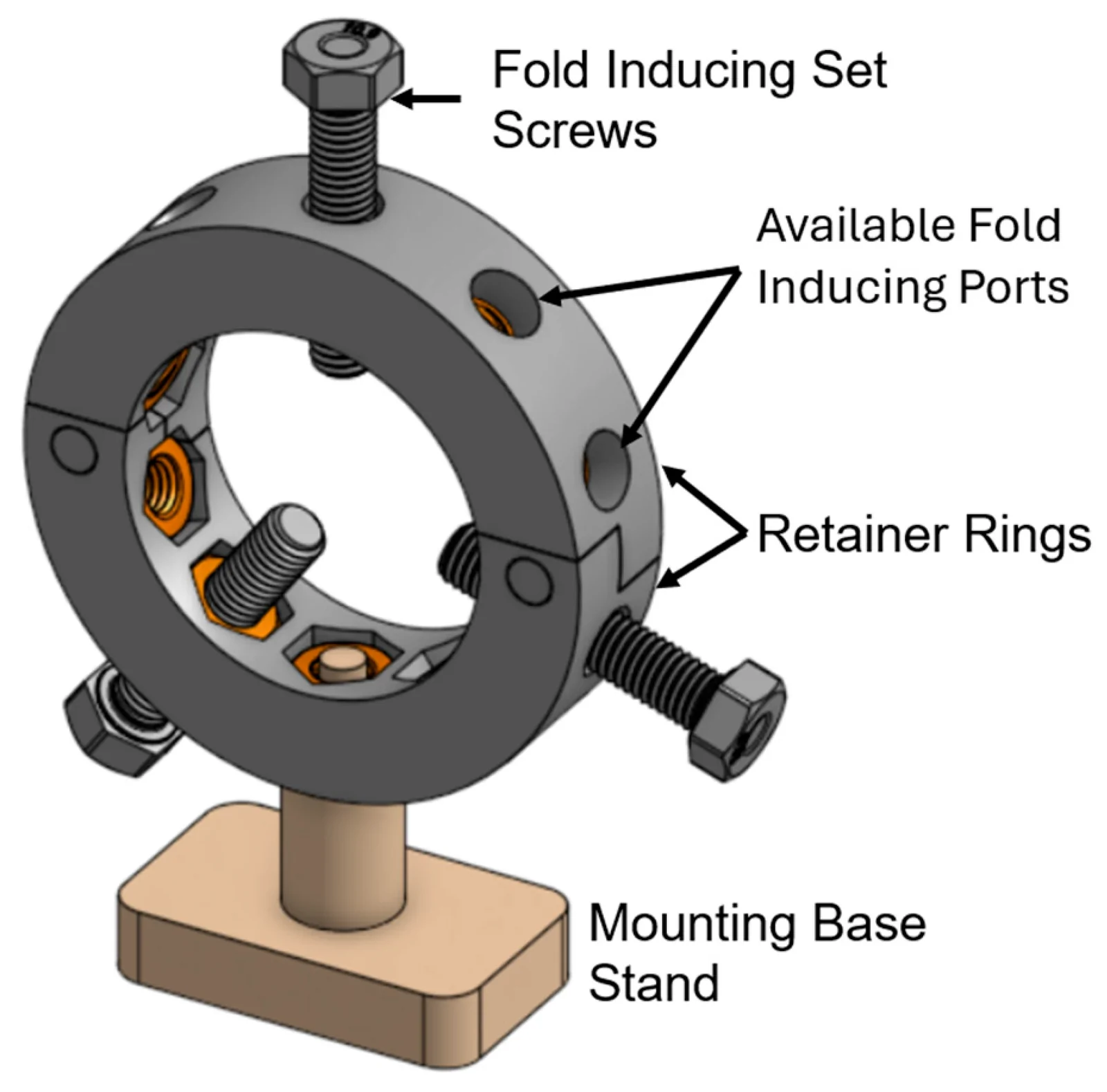
Adjustable retainer-ring assembly for topological shaping. Dual rings mount modularly along the phantom; ten circumferential ports hold fold-inducing set screws for controlled wall indentation, and a base stand supports the phantom during folding and imaging.

To introduce physiologic folding, 3D-printed adjustable retainer rings were attached along the phantom (Figure 6). Each ring incorporated ten circumferential set screws for controlled wall indentation, enabling the precise formation of duodenal-like folds (Figure 5E). The modular design allowed quick repositioning and repeatable fold patterns without structural deformation.

### 2.5. Benchtop Simulation and Optical Validation

**Figure 7:**
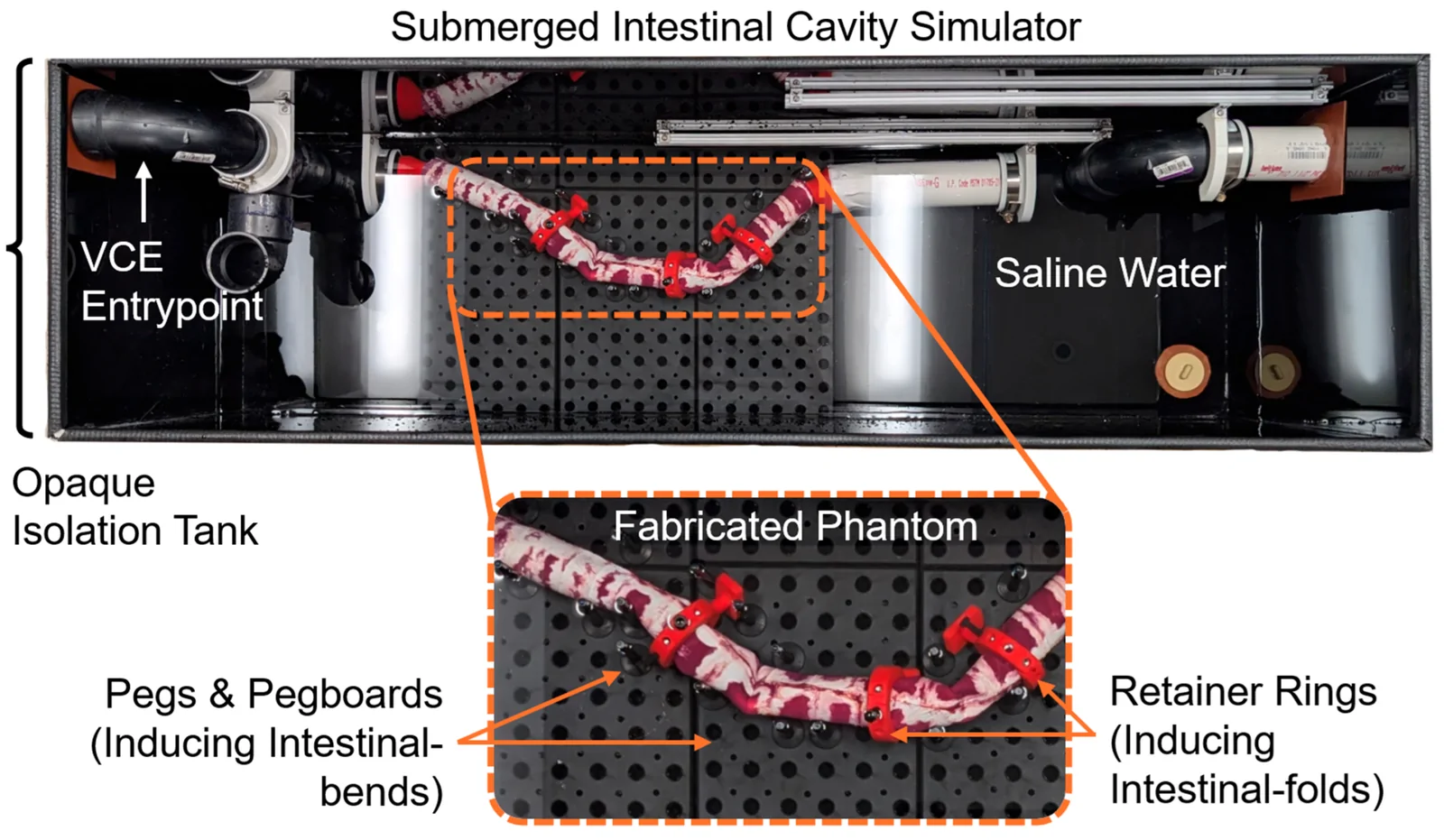
Submerged benchtop intestinal cavity simulator for VCE evaluation. The phantom is mounted on perforated pegboards in an opaque, saline-filled tank; repositionable pegs induce intestinal-like bends and retainer rings impose luminal folds. A dedicated entry point admits the capsule, and a top lid excludes ambient light. Inset: assembled phantom with peg and retainer-ring elements.

Prior to imaging, phantoms were immersed in saline for 12 h to reproduce the wet, hydrated optical environment of the lumen. The phantom was then integrated into a benchtop intestinal cavity simulator designed to reproduce the geometric and optical conditions of the in vivo small bowel (Figure 7) [26]. The phantom was mounted on perforated pegboards using repositionable pegs that constrained its path into intestinal-like bends along its length, while the modular retainer rings described in Section 2.4 imposed localized wall indentations to reproduce physiologic luminal folds. This arrangement allowed the macro-scale bends and folds to be adjusted independently of the villous microstructure, emulating the curvature and looping encountered during small-bowel navigation.

The assembled simulator was submerged in saline within an opaque isolation tank, maintaining the phantom in a hydrated state throughout the imaging process. During acquisition, a top lid sealed the tank to exclude ambient light so that the only illumination reaching the phantom originated from the capsule itself. This replicated the dark, enclosed environment of the intestinal cavity and prevented external light from confounding the optical measurements. A dedicated VCE entry point on the simulator permitted controlled introduction and advancement of the capsule through the phantom lumen.

A Medtronic PillCam SB3 capsule was passed through the phantom under these conditions, and the captured video was used for the qualitative endoscopic comparison and the SSIM-based benchmarking against clinical imagery reported in Section 3.2.

## 3. Results and Discussion

The fabricated villous microstructures were evaluated for dimensional fidelity, patch-to-patch reproducibility, and optical performance under endoscopic imaging. Dimensional measurements underpinning the following analyses were obtained from the high-resolution optical microscopy of each fabricated patch using a uniform image-processing pipeline. Because exhaustive measurement of every pillar across each 80×30 mm patch was impractical, a representative 4×4 sub-region containing 16 pillars was selected at a randomly chosen location on each patch. Binary thresholding was first applied to isolate the pillar base contours within this sub-region and a Hough circle transform was then used to localize the center coordinate $c_{i}$ and base diameter $d_{i}$ of each pillar $i\in \left\{1,\ldots ,\;16\right\}.$ The height $h_{i}$ of each pillar was extracted as the surface elevation at its corresponding circle center, and the edge-to-edge spacing $s_{ij}$ between two adjacent pillars i and j was computed as the Euclidean distance between centers minus the half-diameter of each pillar:

(1) $$s_{ij}=\left\|c_{i}-c_{j}\right\|-\frac{d_{i}+d_{j}}{2}$$

Applied uniformly to 10 independently fabricated patches per Marsh-stage configuration (n=160 pillars per group, sampled from one randomly located 4×4  sub-region per patch), this procedure yielded the per-pillar measurements $\left\{h_{i},\;d_{i},\;s_{ij}\right\}$ that underpin the dimensional dataset summarized in the following subsections, alongside complementary qualitative and quantitative observations from PillCam endoscopic imaging of the assembled phantom.

### 3.1. Microstructural Accuracy and Fidelity

Dimensional fidelity was assessed by comparing the per-pillar measurements $\left\{h_{i},\;d_{i},\;s_{ij}\right\}$ against the CAD design targets $V_{H}$, $V_{D}$ and $V_{S}$ specified in Section 2.1 across the five Marsh-stage configurations. Two complementary metrics were extracted from the dataset described above. The first, the measured mean (μ) and the standard deviation (σ) of each per-pillar quantity pooled across all 160 pillars within a configuration, captured overall fabrication accuracy, corresponding to the realized values of $V_{H},\;V_{D}$ and $V_{S}$. The second metric characterized patch-to-patch reproducibility, the property most relevant to the modular assembly strategy. For each Marsh-stage configuration, a patch-level mean ${\bar{\mu }}_{k}$ was first computed for each patch k∈{1,…, 10} by averaging the 16 per-pillar measurements of the quantity of interest ($V_{H},\;V_{D}$ or $V_{S}$) within that patch. The coefficient of variation was then computed across the resulting set of patch means as follows:

(2) $$CV=\frac{\sigma_{patch}}{\mu_{grand}}\times 100\%$$

where $\mu_{grand}=\frac{1}{10}\sum_{k}{\bar{\mu }}_{k}$ is the grand mean of the ten patch-level means and $\sigma_{patch}$ is their sample standard deviation. Defined this way, *CV* quantifies how much independently fabricated patches deviate from one another, rather than the pillar-to-pillar variability within a single patch, and therefore speaks directly to whether patches can be interchanged within an assembled phantom.

**Figure 8:**
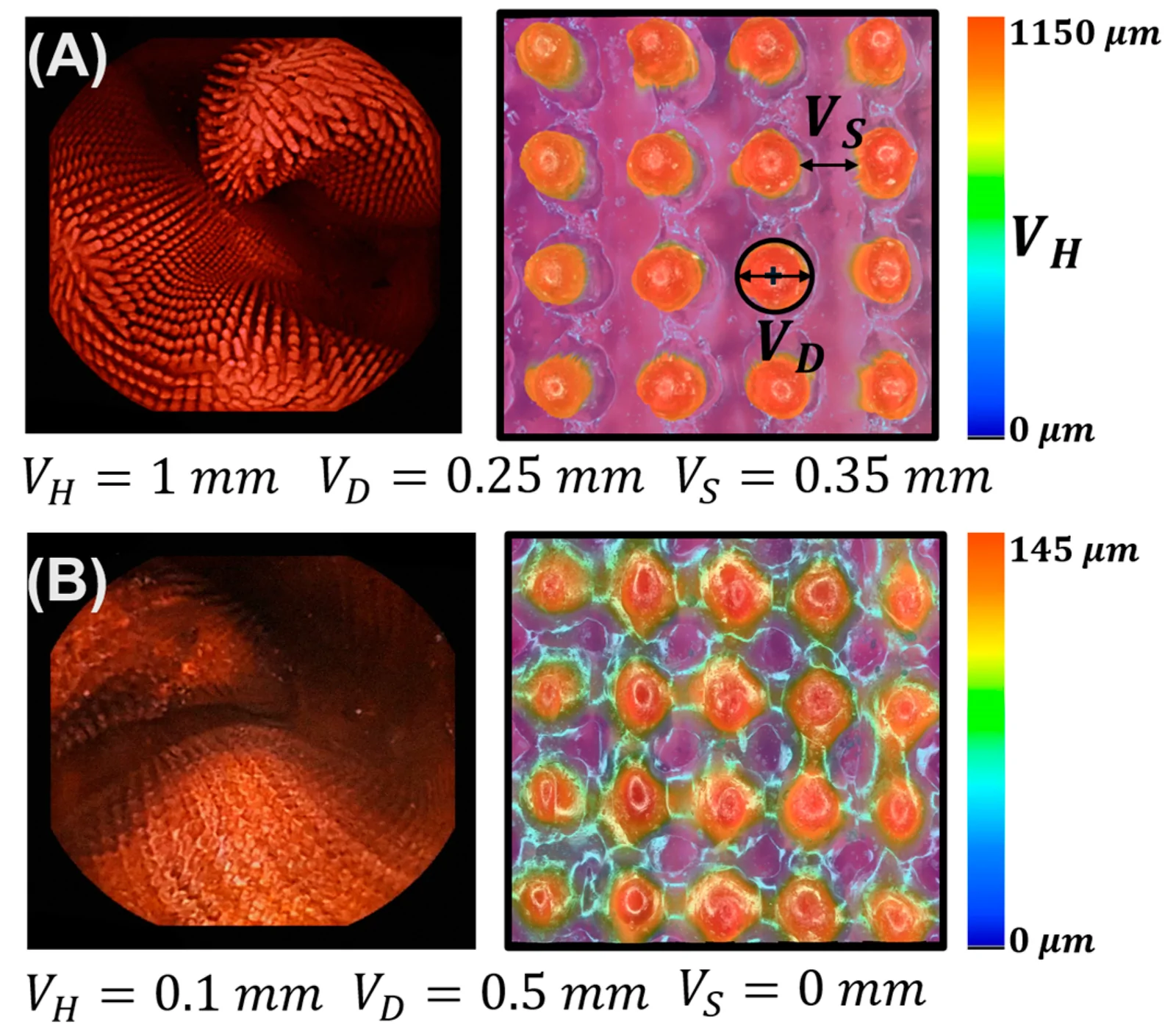
Healthy versus atrophic villous structures in the fabricated phantom. (**A**) Healthy model: tall, closely spaced villi with strong surface texture under endoscopic imaging. (**B**) Atrophic model: short, flattened villi with smoother appearance and reduced contrast. Color maps show the measured villus height distribution.

**Figure 9:**
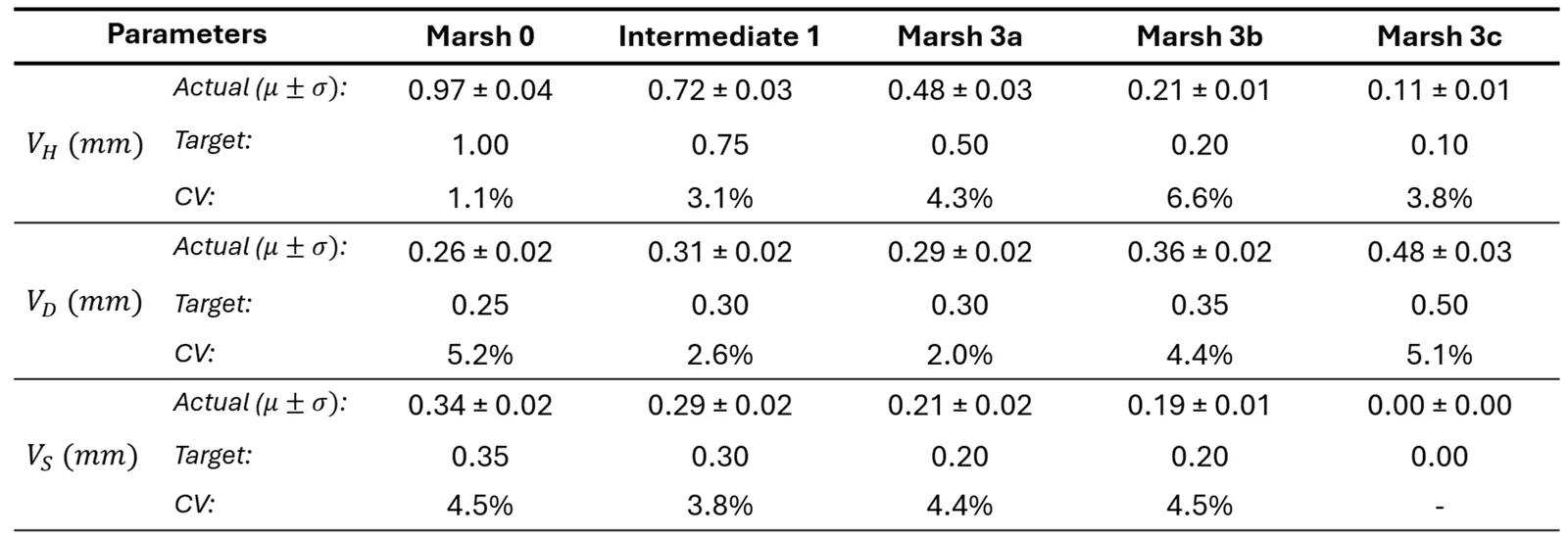
Dimensional fidelity and patch-to-patch reproducibility across Marsh classifications. For each dimension, the measured mean (*μ*) ± SD (*σ*) is shown against the design target, with the coefficient of variation (*CV*) quantifying patch-to-patch reproducibility.

**Figure 10:**
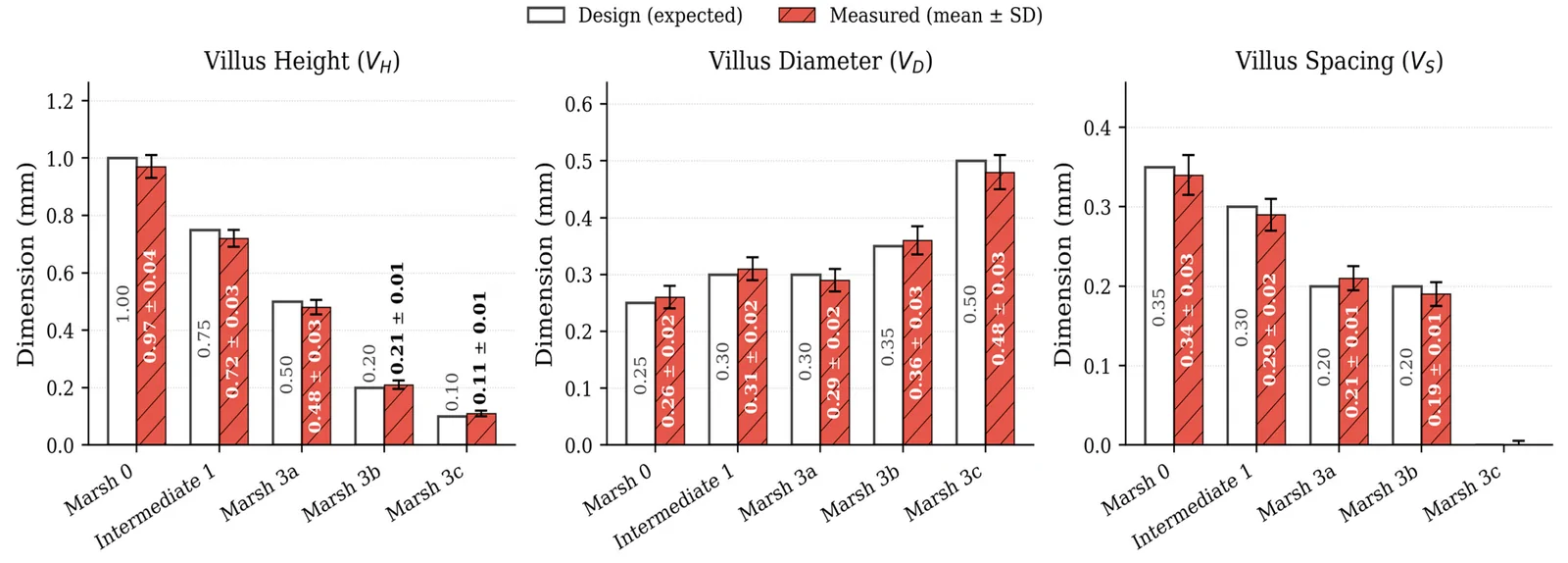
Dimensional accuracy of fabricated villous microstructures. Expected versus measured villus height (*V_H_*), diameter (*V_D_*), and spacing (*V_S_*) across five configurations (Marsh 0–3c); deviations remain within ±10%.

As summarized in Figure 8, Figure 9 and Figure 10, the measured means for $V_{H}$, $V_{D}$, and $V_{S}$ remained within ±10% of their design values across all configurations, and the between-patch *CV* remained below 7% across every dimension and Marsh stage (*CV* is undefined for $V_{S}$ at Marsh 3c, where the design value is zero). Together, these results indicate that the material-jetting process is both accurate (low bias relative to design) and reproducible (low variation across replicates) at sub-millimeter scales. Representative microscopic images of the fabricated patches (Figure 8, right) corroborate the quantitative findings, showing uniform pillar geometry and consistent replication across the printed arrays.

### 3.2. Visual Realism and Optical Performance

Figure 8 compares VCE images captured from the healthy and atrophic phantom configurations. The healthy model ($V_{H}=1.0\;mm,\;V_{D}=0.25\;mm,\;V_{S}=0.35\;mm$) exhibited strong surface texture, natural mucosal sheen, and detailed shadowing, while the atrophic model ($V_{H}=0.1\;mm,\;V_{D}=0.5\;mm,\;V_{S}=0.0\;mm$) appeared smoother with reduced contrast and flattened topology. Together, these contrasting appearances replicate the endoscopic presentation of Marsh 0 and Marsh 3b–3c villous atrophy, respectively. The fabricated height distributions visualized by the PillCam (Figure 8, left) correspond closely to those measured by microscopy (Figure 8, right). Variations in villus height and spacing produced clear differences in apparent optical scattering, evidenced by changes in image brightness, shadowing, and surface contrast under endoscopic illumination.

To complement these qualitative observations with a quantitative measure of visual realism, the phantom was further benchmarked against clinical capsule-endoscopy imagery from the publicly available Kvasir-Capsule dataset [27]. For each of the two extreme villous conditions, 100 PillCam frames were sparsely sampled across the recorded phantom video, and 100 clinical frames of the matching mucosal appearance were drawn from labeled Kvasir-Capsule sequences. Sparse sampling spaced the selected frames across each video to limit the near duplication that consecutive capsule frames exhibit. All phantom imaging was performed under controlled conditions—within a light-sealed, saline-filled tank with the capsule as the sole illumination source and the sample hydrated (Section 2.5)—so that acquisition conditions were held fixed rather than allowed to vary between frames. From each frame, a 100×100 pixel central region of interest was cropped to isolate the mucosal surface from peripheral lens distortion and vignetting, and a Gaussian filter then suppressed the sensor noise. The structural similarity index (SSIM), a perceptually weighted metric that jointly accounts for luminance, contrast, and local structure on a scale from 0 (no similarity) to 1 (identical) [28], was evaluated for every phantom-clinical frame pair, yielding 100 ×100 = 10,000 values per condition. The resulting distribution therefore quantifies aggregate textural correspondence between synthetic and native mucosa rather than pixel-level scene alignment, and averaging the 10,000 values gave a per-condition mean alongside its standard deviation.

The phantom achieved a mean SSIM of 0.625 ± 0.03 against healthy mucosal frames and 0.761 ± 0.01 against severely atrophic mucosal frames, indicating substantial structural correspondence with native tissue appearance under capsule-endoscopy imaging in both conditions. The higher score in the atrophic configuration is consistent with the morphological state of the tissue itself: as villous height collapses and inter-pillar spacing approaches zero, the mucosal surface loses its fine-scale texture and reduces to a comparatively uniform, low-relief field, a regime in which both real and synthetic surfaces present fewer distinguishing structural features and SSIM is correspondingly higher. The lower score in the healthy configuration reflects a genuine limitation of the current fabrication: the printed villi are arranged on a strictly periodic lattice, whereas native intestinal villi exhibit local randomness in position, orientation, and height. This periodicity, while convenient for parametric CAD specification, introduces regular-grid structural cues that real mucosa does not possess and that SSIM correctly penalizes. Future iterations will replace the uniform lattice with a stochastic placement scheme that preserves the literature-derived statistical distributions of villus height, diameter, and spacing while randomizing individual pillar positions, an approach expected to bring the healthy-condition SSIM closer to the atrophic value.

SSIM was adopted as the primary realism metric because it operates in the modality of intended use and, despite its name, provides a quantitative optical rather than purely structural comparison. Following Wang et al. [28], SSIM is the product of luminance, contrast, and structure terms, of which the first two are photometric: the luminance term compares the mean reflected intensity of phantom and clinical frames and therefore reflects diffuse reflectance under the capsule’s illumination, while the contrast term compares intensity variance, which is governed by subsurface scattering and surface relief. The reported values thus quantify the phantom’s optical fidelity—its reflectance and scattering behavior as rendered by the capsule—against native mucosa, and not only its structural resemblance. This image-domain comparison is the appropriate primary measure here because video capsule endoscopy is a white-light, surface-reflectance system: the quantity that governs dataset realism is the integrated optical response the capsule sensor records against real clinical frames, precisely what SSIM evaluates. Bulk optical coefficients, namely the scattering and absorption coefficients obtained by integrating-sphere methods [29], instead describe a material in isolation under controlled spectral illumination. Such coefficients are the primary descriptors for modalities that exploit spectral or volumetric contrast, including hyperspectral reflectance [14] and multimodal optical phantoms [30]. On their own, however, they do not predict the rendered surface appearance that the capsule sensor records, which is the quantity that governs VCE dataset quality.

### 3.3. Modularity and Extensibility

The patch-based fabrication strategy provides a level of flexibility not achievable with monolithic phantom designs. Because each 80 × 30 mm villous patch is fabricated, dyed, and characterized independently before assembly onto the fabric substrate, individual patches representing different Marsh stages can be combined within a single phantom to simulate spatially heterogeneous disease—for example, focal atrophy adjacent to healthy mucosa—as is clinically observed in patchy celiac disease. The fabric substrate length is unconstrained by the printer build volume, allowing the method to scale to phantoms of arbitrary length (duodenum-only, full small bowel, and beyond) by appending additional patches. Damaged or experimentally consumed segments can also be replaced without rebuilding the full construct, supporting reuse and longitudinal studies. This modularity, combined with parametric control of villous geometry at the CAD stage and the demonstrated patch-to-patch reproducibility documented in Section 3.1, makes the framework readily extensible to other tubular mucosal tissues where surface microstructure governs imaging contrast.

The same patch-based principle extends the framework beyond celiac villous atrophy to other focal small-bowel pathologies. Because each patch’s surface geometry and coloration are defined parametrically and fabricated independently, distinct disease features can be encoded as dedicated patch designs: ulcers and erosions as localized surface depressions with darkened or fibrin-toned dyeing, polyps as raised sessile or pedunculated protrusions, and angiectasias as fine red subsurface vascular features produced through multi-material dyeing. High-resolution photopolymer processes have similarly been used to reproduce intestinal surface topographies at comparable scales [31], supporting the feasibility of this extension. Assembling such patches in arbitrary spatial arrangements would allow a single phantom to present mixed findings on a shared substrate. Because these pathologies—like ulcer, polyp, and angiectasia—are underrepresented relative to normal mucosa in clinical capsule-endoscopy datasets [27], a phantom generating balanced, controllable examples of each could help address that data imbalance for imaging and algorithm development.

## 4. Conclusions

This study presents a reproducible and modular fabrication framework for constructing anatomically realistic intestinal phantoms with controlled villous microstructures. By integrating high-resolution material-jetting with a compliant woven substrate and adjustable retainer-ring system, the developed method bridges the gap between digital morphometric modeling and physically verifiable, tissue-like constructs.

The resulting phantom demonstrates high geometric fidelity and visual realism, offering precise control over villus dimensions and topological folding within a hydrated environment. Its modular, patch-based architecture decouples microstructure fabrication from macro-scale assembly, enabling user-defined spatial distributions of disease severity, scalable phantom lengths, and straightforward extension to other tubular mucosal tissues—capabilities that monolithic phantom designs cannot easily provide.

Beyond structural mimicry, this platform establishes a standardized foundation for evaluating capsule-based imaging devices, validating AI-driven diagnostic algorithms, and enhancing clinician training. Future development will pursue four complementary directions. First, the periodic villus lattice will be replaced with a stochastic placement scheme that randomizes individual pillar positions while preserving the literature-derived distributions of villus height, diameter, and spacing, an approach expected to raise the healthy-condition SSIM toward the atrophic value. Second, multi-material fabrication will be introduced to better match the optical and mechanical heterogeneity of native mucosa, moving beyond the single-resin villous layer used here. Third, the mechanical properties of both the villous resin and the woven substrate will be quantitatively characterized against native tissue through tensile and compression testing, and the phantom’s optical coefficients will be measured by integrating-sphere methods, complementing the image-domain validation reported here. Fourth, dynamic peristaltic motion will be incorporated, driven either actively by the robotic actuation platform under development or passively through luminal fluid pressure. Together, these advances will extend the realism and functional scope of the phantom from a static, imaging-focused construct toward a dynamic, multi-property model of the intestinal mucosa.

## Data Availability

Data is available upon reasonable request. Please contact the corresponding author.

## Abbreviations

The following abbreviations are used in this manuscript:

VCE	Video Capsule Endoscopy
GI	Gastrointestinal
CeD	Celiac Disease
CAD	Computer Aided Design
STL	Stereolithography
SSIM	Structural Similarity Index
CV	Coefficient of Variation

## References

[1] Rubio-Tapia A., Hill I.D., Kelly C.P., Calderwood A.H., Murray J.A., American College of Gastroenterology (2013). ACG Clinical Guidelines: Diagnosis and Management of Celiac Disease. Am. J. Gastroenterol..

[2] Adelman D.C., Murray J., Wu T.-T., Mäki M., Green P.H., Kelly C.P. (2018). Measuring Change In Small Intestinal Histology In Patients With Celiac Disease. Am. J. Gastroenterol..

[3] Mocan O., Dumitraşcu D.L. (2016). The Broad Spectrum of Celiac Disease and Gluten Sensitive Enteropathy. Clujul Med..

[4] Hale M.F., Sidhu R., McAlindon M.E. (2014). Capsule Endoscopy: Current Practice and Future Directions. World J. Gastroenterol..

[5] Gora M.J., Sauk J.S., Carruth R.W., Gallagher K.A., Suter M.J., Nishioka N.S., Kava L.E., Rosenberg M., Bouma B.E., Tearney G.J. (2013). Tethered Capsule Endomicroscopy Enables Less-Invasive Imaging of Gastrointestinal Tract Microstructure. Nat. Med..

[6] Vedaei S.S., Wahid K.A. (2021). A Localization Method for Wireless Capsule Endoscopy Using Side Wall Cameras and IMU Sensor. Sci. Rep..

[7] Stoleru C.-A., Dulf E.H., Ciobanu L. (2022). Automated Detection of Celiac Disease Using Machine Learning Algorithms. Sci. Rep..

[8] Sharif K., David P., Omar M., Sharif Y., Patt Y.S., Klang E., Lahat A. (2023). Deep Learning in Coeliac Disease: A Systematic Review on Novel Diagnostic Approaches to Disease Diagnosis. J. Clin. Med..

[9] Li D., Cave D., Li A., Li S. (2024). Enhanced Accuracy for Classification of Video Capsule Endoscopy Images Using Multiple Deep Learning Convolutional Neural Networks. iGIE.

[10] Cloonan A.J., Shahmirzadi D., Li R.X., Doyle B.J., Konofagou E.E., McGloughlin T.M. (2014). 3D-Printed Tissue-Mimicking Phantoms for Medical Imaging and Computational Validation Applications. 3D Print. Addit. Manuf..

[11] Wang K., Ho C.-C., Zhang C., Wang B. (2017). A Review on the 3D Printing of Functional Structures for Medical Phantoms and Regenerated Tissue and Organ Applications. Engineering.

[12] Higgins M., Leung S., Radacsi N. (2022). 3D Printing Surgical Phantoms and Their Role in the Visualization of Medical Procedures. Ann. 3D Print. Med..

[13] Lei J., Tee L.B.G., Ragunath K., Sun Z. (2025). Three-Dimensional-Printed Gastrointestinal Tract Models for Surgical Planning and Medical Education: A Systematic Review. Appl. Sci..

[14] Wang J., Ghassemi P., Melchiorri A., Ramella-Roman J., Mathews S.A., Coburn J., Sorg B.S., Chen Y., Pfefer J. (2015). 3D Printed Biomimetic Vascular Phantoms for Assessment of Hyperspectral Imaging Systems. Proceedings of the Design and Performance Validation of Phantoms Used in Conjunction with Optical Measurement of Tissue VII.

[15] Zulina N., Caravaca O., Liao G., Gravelyn S., Schmitt M., Badu K., Heroin L., Gora M.J. (2021). Colon Phantoms with Cancer Lesions for Endoscopic Characterization with Optical Coherence Tomography. Biomed. Opt. Express.

[16] Das P., Gahlot G.P., Singh A., Baloda V., Rawat R., Verma A.K., Khanna G., Roy M., George A., Singh A. (2019). Quantitative Histology-Based Classification System for Assessment of the Intestinal Mucosal Histological Changes in Patients with Celiac Disease. Intest. Res..

[17] Vargas M.M., Artigiani Neto R., Sdepanian V.L. (2022). Quantitative Histology as a Diagnostic Tool for Celiac Disease in Children and Adolescents. Ann. Diagn. Pathol..

[18] Taavela J., Viiri K., Välimäki A., Sarin J., Salonoja K., Mäki M., Isola J. (2021). Apolipoprotein A4 Defines the Villus-Crypt Border in Duodenal Specimens for Celiac Disease Morphometry. Front. Immunol..

[19] Ehsan L., Coomes D., Kelly P., Greene A.R., Ali S.A., Mulenga C., Denno D.M., VanBuskirk K., Raghib M.F., Mahfuz M. (2024). Duodenal Quantitative Mucosal Morphometry in Children with Environmental Enteric Dysfunction: A Cross-Sectional Multicountry Analysis. Am. J. Clin. Nutr..

[20] Marsh M.N. (1992). Gluten, Major Histocompatibility Complex, and the Small Intestine. A Molecular and Immunobiologic Approach to the Spectrum of Gluten Sensitivity (‘celiac Sprue’). Gastroenterology.

[21] Oberhuber G., Granditsch G., Vogelsang H. (1999). The Histopathology of Coeliac Disease: Time for a Standardized Report Scheme for Pathologists. Eur. J. Gastroenterol. Hepatol..

[22] Materials For Manufacturing. https://www.stratasys.com/en/stratasysdirect/materials/.

[23] Krause M., Marshall A., Catterlin J.K., Hornik T., Kartalov E.P. (2024). Dimensional Fidelity and Orientation Effects of PolyJet Technology in 3D Printing of Negative Features for Microfluidic Applications. Micromachines.

[24] Emiliani N., Porcaro R., Pisaneschi G., Bortolani B., Ferretti F., Fontana F., Campana G., Fiorini M., Marcelli E., Cercenelli L. (2024). Post-Printing Processing and Aging Effects on Polyjet Materials Intended for the Fabrication of Advanced Surgical Simulators. J. Mech. Behav. Biomed. Mater..

[25] Bochnia J. (2023). A Study of the Mechanical Properties of Naturally Aged Photopolymers Printed Using the PJM Technology. Materials.

[26] Dey R., Martin J., Du J., Siegelman J., Hacunda J., Topic S., Yang C., Zheng Y. (2026). A Robotic Wire-Driven Video Capsule Endoscopy Platform for Controlled Data Generation in Gastrointestinal Diagnostics. IEEE Trans. Med. Robot. Bionics.

[27] Smedsrud P.H., Thambawita V., Hicks S.A., Gjestang H., Nedrejord O.O., Næss E., Borgli H., Jha D., Berstad T.J.D., Eskeland S.L. (2021). Kvasir-Capsule, a Video Capsule Endoscopy Dataset. Sci. Data.

[28] Wang Z., Bovik A.C., Sheikh H.R., Simoncelli E.P. (2004). Image Quality Assessment: From Error Visibility to Structural Similarity. IEEE Trans. Image Process..

[29] Wei H.-J., Xing D., Lu J.-J., Gu H.-M., Wu G.-Y., Jin Y. (2005). Determination of Optical Properties of Normal and Adenomatous Human Colon Tissues in Vitro Using Integrating Sphere Techniques. World J. Gastroenterol..

[30] Smith G.T., Lurie K.L., Zlatev D.V., Liao J.C., Ellerbee Bowden A.K. (2016). Multimodal 3D Cancer-Mimicking Optical Phantom. Biomed. Opt. Express.

[31] Creff J., Courson R., Mangeat T., Foncy J., Souleille S., Thibault C., Besson A., Malaquin L. (2019). Fabrication of 3D Scaffolds Reproducing Intestinal Epithelium Topography by High-Resolution 3D Stereolithography. Biomaterials.

